# Supplementary material for: Bacterial cell cycle control by citrate synthase independent of enzymatic activity
Source: eLife. 2020 Mar 9;9:e52272. doi: 10.7554/eLife.52272 (PMC7083601; doi:10.7554/eLife.52272)
Supplement: Supplementary file 1. [file elife-52272-supp1.docx]

**Supplementary File 4 – Table of *C. crescentus* and *E. coli* strains**

| Name | Relevant genotype/ description | source or ref |
| --- | --- | --- |
| ***C. crescentus* strains** | | |
| MB1 | NA1000; Synchronizable derivative of wild-type strain CB15 | (Evinger and Agabian 1977) |
| MB656 | NA1000; *spmX*::*spmX-mCherry* | (Radhakrishnan et al. 2008) |
| UG430 | NA1000; *stpX* ::*stpX-GFP*; *spmX*::*spmX-mCherry* | This study |
| MB557 | NA1000; *parB*::*GFP*-*parB* | (Thanbichler and Shapiro 2006) |
| MB2403 | NA1000 ; ∆*spoT* | (Boutte et al. 2012) |
| MB2417 | NA1000 ; ∆*ptsP* | (Sanselicio and Viollier 2015) |
| MB556 | NA1000; *∆tipN* | (Huitema et al. 2006) |
| MB46 | NA1000; *∆popA* | (Duerig et al. 2009) |
| MB47 | NA1000; *∆cpdR::*Ω | (Iniesta et al. 2006) |
| MB48 | NA1000; *∆rcdA::hyg* | (McGrath et al. 2006) |
| MB2001 | NA1000; *∆cpdR::tet* | (Skerker et al. 2005) |
| MB1972 | NA1000 ; ∆*kidO* ; *xylX* ::*kidO*^AA::DD^ | (Radhakrishnan et al. 2010) |
| MB2405 | NA1000 ; ∆*kidO* | (Radhakrishnan et al. 2010) |
| MB3282 | NA1000 ; *xylX ::relA’-flag* | (Gonzalez and Collier 2014) |
| MB3288 | NA1000 ; *xylX ::relA’^E335Q^-flag* | (Gonzalez and Collier 2014) |
| MB3075 | NA1000; ∆*tipN*; ∆*popA* | This study |
| MB3079 | NA1000; ∆*tipN*; ∆*rcdA::*Ω | This study |
| MB2017 | NA1000; ∆*tipN*; ∆*cpdR::tet* | This study |
| MB2366 | NA1000 ; ∆tipN; *xylX* ::*kidO*^AA::DD^ | This study |
| MB2720 | NA1000; ∆*tipN* ; ∆*cpdR ::tet*; ∆*kidO* | This study |
| MB2325 | NA1000; *pilA*::*P_pilA_*-*GFP* | This study |
| MB2327 | NA1000; ∆*cpdR::*Ω; *pilA*::*P_pilA_*-*GFP* | This study |
| MB2329 | NA1000; ∆*tipN*; *pilA*::*P_pilA_*-*GFP* | This study |
| MB2331 | NA1000; ∆*tipN*; ∆*cpdR::*Ω; *pilA*::*P_pilA_*-*GFP* | This study |
| MB2268 | NA1000; *pilA*::*P_pilA_*-*nptII* | This study |
| MB2271 | NA1000; ∆*tipN*; ∆*cpdR::tet*; *pilA*::*P_pilA_*-*nptII* | This study |
| MB3056 | NA1000; ∆*tipN*; ∆*cpdR::tet*; *citA*::*Tn*; *pilA*::*P_pilA_*-*nptII* | This study |
| MB3058 | NA1000; ∆*tipN*; ∆*cpdR::tet*; ∆*citA*; *pilA*::*P_pilA_*-*nptII* | This study |
| MB2679 | NA1000; ∆*citB*; ∆*citC* | This study |
| MB2622 | NA1000; *citA*::*Tn* | This study |
| MB2559 | NA1000; ∆*citA; citA*::pNTPS138-∆*citA* | This study |
| MB1537 | NA1000; pMT335 | This study |
| MB3433 | NA1000 ; ∆*citA*; pMT335 | This study |
| MB3435 | NA1000; ∆*citA*; pMT335-*citA* | This study |
| MB3469 | NA1000; ∆*citA*; pMT335-*citB* | This study |
| MB3471 | NA1000; ∆*citA*; pMT335-*citC* | This study |
| MB3473 | NA1000 ; ∆*citA*; pMT335-*gltA* | This study |
| MB3437 | NA1000; ∆citA; pMT335-citA^H303W^ | This study |
| MB3439 | NA1000; ∆citA; pMT335-citA^H303A^ | This study |
| MB2452 | NA1000; *parB*::*GFP*-*parB*; *citA*::*Tn* | This study |
| MB3467 | NA1000; *parB*::*GFP*-*parB*; ∆*citA; citA ::pNTPS138-∆citA* | This study |
| MB2413 | NA1000 ; ∆*spoT* ; *citA:*:*Tn* | This study |
| MB2426 | NA1000 ; ∆*ptsP* ; *citA:*:*Tn* | This study |
| MB3601 | NA1000 ; ∆*popA;* plac290-*P_pilA_* | This study |
| MB3605 | NA1000 ; ∆*popA;* plac290-*P_hfsJ_* | This study |
| MB3607 | NA1000 ; ∆*cpdR;* plac290-*P_pilA_* | This study |
| MB3611 | NA1000 ; ∆*cpdR;* plac290-*P_hfsJ_* | This study |
| MB3623 | NA1000 ; ∆*citA;* plac290-*P_pilA_* | This study |
| MB3625 | NA1000 ; ∆*citA;* plac290-*P_fljM_* | This study |
| MB3627 | NA1000 ; ∆*citA;* plac290-*P_ctrA_* | This study |
| MB3590 | NA1000 ; ∆*citA;* plac290-*P_sciP_* | This study |
| MB3592 | NA1000 ; ∆*citA;* plac290-*P_hfsJ_* | This study |
| MB3594 | NA1000 ; ∆*citA;* plac290-*P_ccrM_* | This study |
| MB3596 | NA1000 ; ∆*citA;* plac290-*P_fliQ_* | This study |
| MB3615 | NA1000*; citA*::Tn spmX*::spmX-mCherry; xylX::mipZ-YFP* | This study |
| MB3568 | NA1000*; spmX::spmX-mCherry; xylX::mipZ-YFP* | This study |
| MB3598 | NA1000 ; ∆citA ::Tn, *stpX* ::*stpX-GFP*; *spmX*::*spmX-mCherry* | This study |
| MB3566 | NA1000*; spmX::spmX-mCherry; tipF::tipF-GFP* | This study |
| MB3613 | NA1000*;* citA::Tn spmX*::spmX-mCherry; tipF::tipF-GFP* | This study |
| ***E. coli* strains** |  |  |
| S17-1 | RP4, Tc::Mu Km::Tn7 | (Simon et al. 1983) |
| EC100D | F-mcrA Δ(mrr-hsdRMS-mcrBC) Φ80dlacZΔM15 ΔlacX74 recA1 endA1 araD139 Δ(ara, leu)7697 galU galK λ-rpsL (StrR) nupG | Epicentre |
| BW25113 | F-Δ(araD-araB)567ΔlacZ4787(::rrnB-3)rph-1Δ(rhaD-rhaB)568hsdR514 | CGSC |
| JW0710-1 | BW35113; Δ*gltA770::kan* | (Baba et al. 2006) |
| eMB554 | BW35113; pMT335 | This study |
| eMB556 | BW35113; ∆*gltA770::kan*; pMT335 | This study |
| eMB558 | BW35113; ∆*gltA770::kan*; pMT335-*citA* | This study |
| eMB560 | BW35113; ∆*gltA770::kan*; pMT335-*citB* | This study |
| eMB562 | BW35113; ∆*gltA770::kan*; pMT335-*citC* | This study |
| eMB564 | BW35113; ∆*gltA770::kan*; pMT335-*gltA* | This study |
| eMB581 | BW35113; ∆*gltA770::kan*; pMT335-*citA^H303W^* | This study |
| eMB583 | BW35113; ∆*gltA770::kan*; pMT335-*citA^H303A^* | This study |
|  |  |  |
