# Supplementary material for: Bacterial cell cycle control by citrate synthase independent of enzymatic activity
Source: eLife. 2020 Mar 9;9:e52272. doi: 10.7554/eLife.52272 (PMC7083601; doi:10.7554/eLife.52272)
Supplement: Supplementary file 2. [file elife-52272-supp2.docx]

**Supplementary File 5 – Table of Plasmids**

| Name | description | source or ref |
| --- | --- | --- |
|  | | |
| pNTPS138 | Two-part selection in in-frame deletion- integration vector: oriT+ sacB+ KanR | Alley MRK, unpublished |
| pMT335 | High copy plasmid carrying a PVan promoter (gentR) | (Thanbichler et al. 2007) |
| pXGFPN-2 | Integration of C-terminal egfp-fusions at Caulobacter P_xylx_ locus (kanR) | (Thanbichler et al. 2007) |
| pSC | Derivative of pET26b (kanR) | (Bergé et al. 2016) |
| plac290-P*_fljM_*  plac290-P*_ctrA_*  plac290-P*_sciP_*  plac290-P*_hfsJ_*  plac290-P*_pilA_*  plac290-P*_fliQ_*  plac290-P*_ccrM_*  pMB278 | Promoter probe plasmid with P*_fljM_*-*lacZ*  Promoter probe plasmid with P*_ctrA_*-*lacZ*  Promoter probe plasmid with P*_sciP_*-*lacZ*  Promoter probe plasmid with P*_hfsJ_*-*lacZ*  Promoter probe plasmid with P*_pilA_*-*lacZ*  Promoter probe plasmid with P*_fliQ_*-*lacZ*  Promoter probe plasmid with P*_ccrM_*-*lacZ*  pNTPS138-∆*citA* | (Fumeaux et al, 2014)  (Fumeaux et al, 2014)  (Fumeaux et al, 2014)  (Fumeaux et al, 2014)  (Skerker et al, 2000)  (Fumeaux et al, 2014)  (Stephens et al, 1995)  This study |
| pMB288 | pNTPS138-∆*citB* | This study |
| pMB289 | pNTPS138-∆*citC* | This study |
| pMB309 | pNTPS138-∆*citB/C* | This study |
| pMB302 | pMT335-*citA* | This study |
| pMB303 | pMT335-*citB* | This study |
| pMB304 | pMT335-*citC* | This study |
| pMB310 | pMT335-*gltA* | This study |
| pMB287 | pSC-*citA* | This study |
| pMB325 | pMT335-*citA^H303W^* | This study |
| pMB326 | pMT335-*citA^H303A^* | This study |
|  |  |  |
