# Supplementary material for: Bacterial cell cycle control by citrate synthase independent of enzymatic activity
Source: eLife. 2020 Mar 9;9:e52272. doi: 10.7554/eLife.52272 (PMC7083601; doi:10.7554/eLife.52272)
Supplement: Supplementary file 3. [file elife-52272-supp3.docx]

**Supplementary File 6 – Table of Oligonucleotides**

| Name | sequence |  |
| --- | --- | --- |
|  | | |
| OMB173 | 5’- aaaaaaagcttTGGACTGGGCCAAGCTCAATC -3’ |  |
| OMB174 | 5’- aaaaaCTGCAGATCGTCAGCGTGGC -3’ |  |
| OMB175 | 5’- aaaaaCTGCAGCTACGTCACGCTCGACAAG -3’ |  |
| OMB176 | 5’- aaaaagaattcGTGCATGCCATGGTCGTGCTC -3’ |  |
| OMB184 | 5’- aaaaaaagcttTCAGCCAGGGTCAGGAAC -3’ |  |
| OMB185 | 5’- aaaaaCATATGGGCGATCACGCCCTCAAGAC -3’ |  |
| OMB186 | 5’- aaaaaCATATGGTCGGACCCGAGGTC -3’ |  |
| OMB187 | 5’- aaaaagaattcATCGCCCGAGATCGCG -3’ |  |
| OMB188 | 5’- aaaaaaagcttACGCCTCGGATCATCCGCAG -3’ |  |
| OMB189 | 5’- aaaaaCATATGCAGAACCTGCTCTGCGTC -3’ |  |
| OMB190 | 5’- aaaaaCATATGGCGGGCTACTCGCCCTC -3’ |  |
| OMB191 | 5’- aaaaagaattcTTGTCGGTCGCGCAGTTC -3’ |  |
| OMB179 | 5’- aaaaacatATGACCGATAAAGCCACGCTG -3’ |  |
| OMB182 | 5’- aaaaagaattcAGCGCTTGTCGAGCGTGACG -3’ |  |
| OMB194 | 5’- aaaaaCATATGATGTCTGATGGTCTTGAGGGCGTG -3’ |  |
| OMB195 | 5’- aaaaagaattcaAGCCGCGACGCGGACCTC -3’ |  |
| OMB196 | 5’- aaaaaCATATGACCGACTGGATGGACG -3’ |  |
| OMB197 | 5’- aaaaagaattcaTGAGGATGAGGAGGGCGAG -3’ |  |
| OMB203 | 5’- aaaaacatATGGCTGATACAAAAGCAAAACTC -3’ |  |
| OMB204 | 5’- aaaaagaattcAACGCTTGATATCGCTTTTAAAGTC -3’ |  |
| OMB183 | 5’- aaaaaaagcttGCGCTTGTCGAGCGTGAC -3’ |  |
| OMB232 | 5’- ctgatgggcttcggctggCGCGTGTACAAGAAC -3’ |  |
| OMB233 | 5’- GTTCTTGTACACGCGccagccgaagcccatcag -3’ |  |
| OMB236 | 5’- CTGATGGGCTTCGGCgccCGCGTGTACAAGAAC -3’ |  |
| OMB237 | 5’- GTTCTTGTACACGCGggcGCCGAAGCCCATCAG-3’ |  |
