# Supplementary material for: Bacterial cell cycle control by citrate synthase independent of enzymatic activity
Source: eLife. 2020 Mar 9;9:e52272. doi: 10.7554/eLife.52272 (PMC7083601; doi:10.7554/eLife.52272)
Supplement: Supplementary file 4. [file elife-52272-supp4.docx]

This file contains information om reagents and antibodies used in the study by Bergé et al

| **Key Resources Table** | | | | |
| --- | --- | --- | --- | --- |
| **Reagent type (species) or resource** | **Designation** | **Source or reference** | **Identifiers** | **Additional information** |
| Strain, Strain Background (*Caulobacter crescentus* NA1000) | *Caulobacter* *crescentus* NA1000 | Evinger and Agabian; PMID: 334726 |  | See table of strains |
| Antibody | CtrARabbit polyclonal | Delaby, 2019 PMID: **31598724** | CtrA antibody are home-made  raised against the full-length protein of *C. crescentus*. | Immunoblot: 1/5000 dilution |
| Antibody | CitARabbit polyclonal | This study | CitA antibody are home-made raised against the full-length protein of *C. crescentus.* | immunoblot: 1/5000 dilution  Description in the material and methods section |
| Antibody | E. coli RNA Polymerase Antibody Sampler KitMouse monoclonal | Biolegend | 699907 | Mix 1:1:1:1 between all sera.  ChIP-Seq: 1/500 dilution |
| commercial assay or kit | Phos-tag | FUJIFILM Wako Chemicals | Distributor  300-93523  Manufacturer  AAL-107M | 25 μM final |
| Chemical Compound | H332PO4 | Hartmann Analytic | cat n° P-RB-1 |  |
| Chemical Compound | Acetonitrile OPTIMA LC/MS Grade | Fisher Scientific | A955-212 |  |
| Chemical Compound | Methanol OPTIMA LC/MS Grade | Fisher Scientific | A456-212 |  |
| chemical compound | Water LC/MS Grade | Fisher Scientific | W/0112/17 |  |
| Chemical Compound | Formic Acid | Biosolve | 069141 |  |
| Chemical Compound | Ammonium hydroxide solution 25% | Sigma-Aldrich | 30501 |  |
| Chemical Compound | Mass Spectrometry Metabolite Library | Sigma-Aldrich | MSMLS-1EA |  |
| Chemical Compound | Major Mix IMS TOF calibration kit | Waters | 186008113 |  |
| Chemical Compound | Leucine Enkephalin | Waters | 700004768-1 | Waters TOF G2-S Sample Kit -2 (700008892) |
| Software, Algorithm | UNIFI V.1.9.3 | Waters |  |  |
| Software, Algorithm | Progenesis QI v2.3 | Nonlinear Dynamics, Waters |  |  |
| Software, Algorithm | SIMCA-P 15.0 | Umetrics |  |  |
| Software, Algorithm | MicrobeJ | Ducret, 2017 PMID: 27572972 |  |  |
| Software, Algorithm | SeqMonk | Babraham bioinformatics institute | V1.40.0 |  |
| Other | polyethyleneimine (PEI) plate | Sigma-Aldrich | Ref Z122882-25EA |  |
| Other | Merck SeQuant ZIC-pHILIC column (150 x 2.1 mm, 5 µm) | Merck Millipore | 1504600001 |  |
| Other | Merck SeQuant ZIC-pHILIC Guard kit (20 x 2.1 mm, 5 µm) | Merck Millipore | 1504380001 |  |
